# Enhanced gastric retention and drug release *via* development of novel floating microspheres based on Eudragit E100 and polycaprolactone: synthesis and *in vitro* evaluation

**DOI:** 10.1080/15685551.2017.1326702

**Published:** 2017-05-10

**Authors:** Umar Farooq, Samiullah Khan, Shahid Nawaz, Nazar Mohammad Ranjha, Malik Salman Haider, Muhammad Muzamil Khan, Eshwa Dar, Ahmad Nawaz

**Affiliations:** ^a^ Faculty of Pharmacy, Bahauddin Zakariya University, Multan, Pakistan; ^b^ Faculty of Pharmacy and Alternative Medicine, The Islamia University of Bahawalpur, Bahawalpur, Pakistan; ^c^ Islam College of Pharmacy, Sialkot, Pakistan; ^d^ Department of Pharmacy, COMSATS Institute of Information Technology, Abbottabad, Pakistan

**Keywords:** Floating microspheres, metronidazole benzoate, oil in water (o/w) method, gastric retention and drug release, Eudragit E 100

## Abstract

Eudragit E 100 and polycaprolactone (PCL) floating microspheres for enhanced gastric retention and drug release were successfully prepared by oil in water solvent evaporation method. Metronidazole benzoate, an anti-protozoal drug, was used as a model drug. Polyvinyl alcohol was used as an emulsifier. The prepared microspheres were observed for % recovery, % degree of hydration, % water uptake, % drug loading, % buoyancy and % drug release. The physico-chemical properties of the microspheres were studied by calculating encapsulation efficiency of microspheres and drug release kinetics. Drug release characteristics of microspheres were studied in simulated gastric fluid and simulated intestinal fluid i.e., at pH 1.2 and 7.4 respectively. Fourier transform infrared spectroscopy was used to reveal the chemical interaction between drug and polymers. Scanning electron microscopy was conducted to study the morphology of the synthesized microspheres.

## 1. Introduction

Drugs with short half are eliminated very abruptly after a brief time in the body hence they require multiple dosing [1,2]. A problem frequently encountered with conventional controlled release dosage forms is their inability to increase their residence time in the stomach and proximal portion of the small intestine. Retention of drug delivery systems in the stomach prolongs overall gastrointestinal transit time, thereby, resulting in improved oral bioavailability of the basic drugs that have poor solubility in higher pH, as well as, drugs susceptible to circadian variations [3]. Various approaches have been developed to retain the drugs in the stomach. These methods mainly include Floating drug delivery system (FDDS), swelling and expanding systems, modified-shape systems, polymeric mucoadhesive systems, high-density systems, and other delayed gastric emptying devices are few techniques to prolong the gastric residence time of drugs [4]. Gastro-retentive drug delivery system which is also known as FDDS is one of the most important techniques developed until now to retain the drug for a longer period of time in the stomach. They increase the gastric retention time (GRT) without affecting the intrinsic rate of gastric emptying. They have a bulk density less than the gastric medium, due to their less density they float in the gastric medium [5].

But unfortunately, single-unit based floating devices (also known as hydrodynamically balanced systems are unreliable in enhancing the GRT due to their ‘all-or-nothing’ emptying process. This phenomena may lead to high variability in the bioavailability and local irritation occur due to a large release at a specific site of the gastrointestinal tract (GIT) [6].

However In contrast, multiple unit particulate dosage forms (e.g., microspheres) can pass uniformly through the GIT and have the added advantages that they can avoid the vagaries of gastric emptying and provide an controlled release, lead to the reduction of intersubjective variability in absorption and local irritation [7].

FDDS is best choice for the drugs that act locally in the stomach or absorbed at acidic pH, drugs which are unstable at alkaline pH, insoluble or poorly soluble at alkaline pH and drugs having narrow therapeutic windows [8]. Floating microspheres have a density less than gastric content i.e., less than 1.004 g/cm³, due to less density they float easily in the stomach. The drug is released at controlled rate while the microspheres float in the gastric fluid. Microspheres can remain floated or buoyant in the stomach for more than 10 h [9].

Poly (e-caprolactone) (PCL) is one of the biocompatible and biodegradable aliphatic polyester synthetic polymer which is approved Food and Drug Administration. It is aliphatic polyester which is biodegradable and biocompatible [10–12]. PCL is prepared from *ε*-caprolactone that has a cyclic structure. In the presence of a suitable catalyst ring opening polymerization takes place [13,14]. 2-methylene-1-3-dioxepane may also prepare PCL by free radical ring opening polymerization [15]. PCL is a crystalline polymer with melting point 59–64 °C. It is hydrophobic polymer [16,17]. It is used as biomaterial for human bodies. It may be used in sutures, drug delivery devices, wound dressings and adhesion barrier. PCL have been used in various sustained release dosage forms and targeted drug delivery systems [18]. PCL is also used in cutaneous wound dressings [19] and release vehicle for chlorhexidine [20].

Eudragit E 100 is a cationic polymer having dimethyl aminoethyl methacrylate as a functional group. Eudragit E 100 is pH dependent polymer, soluble in gastric fluid and swells at pH lower than 5.0. It has good adhesion, low viscosity and high pigment biding. It is film/insulting coating material which is used for taste/odour masking and light/moisture protection [21]. Eudragit E100 is used is transdermal drug delivery system as it produces a good adhesive transparent film [22]. Eudragit E 100 is used in gene therapy for treating hereditary disease. Autoimmune diabetes can be prevented with nanoparticles produced by blending polylactic glycolic acid and Eudragit E 100 for plasmid delivery [23,24].

**Figure 1.**
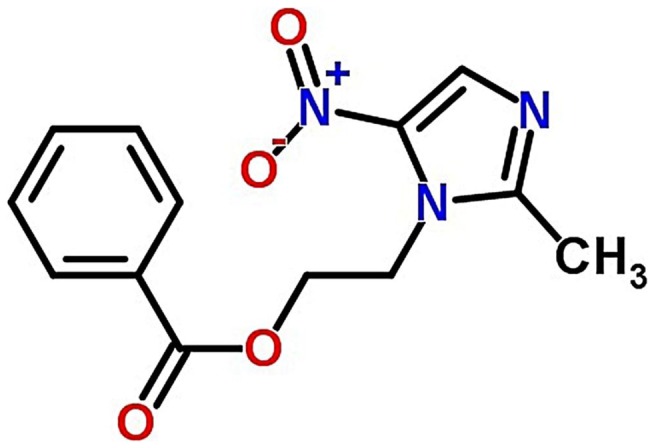
The chemical structure of metronidazole benzoate (MZB).

Current work focus on the design of floating/hollow microspheres to increase the GRT and in turn sustained release of model drug i.e., metronidazole benzoate (MZB) in the stomach by using blend of E100 and PCL in the presence of polyvinyl alcohol (PVA) as an emulsifier. Microspheres were prepared by solvent evaporation method and drug was loaded by *in situ* loading method i.e., during the manufacturing process. Microspheres were characterized for hydration and recovery properties. Effect of different polymer ratio i.e., PCL and E100 on *in vitro* drug release, drug entrapment efficiency, drug loading and drug release kinetics was investigated. Drug release was checked at simulated gastric fluid pH 1.2 as well as at pH 7.4 and then pharmacokinetics models were applied for confirmation of drug release mechanism. Rheological studies were carried out to investigate the flow properties of microspheres. Buoyancy of microspheres in the simulated gastric fluid was assessed. Scanning electron microscopy (SEM) was used to determine the size ranges and morphology of microspheres. Fourier Transform Infrared spectroscopy (FT-IR) was carried out to determine the cross linking and interaction between polymers with drug and without drug loading. Figure 1 indicates the chemical structure of MZB.

## 2. Experimental

### 2.1. Materials

MZB (Purity 99.8%) was a gift received from Siza International (pvt) Ltd. Lahore, Pakistan. Eudragit E 100 (Evonik, Germany) (Purity 99%). Polycaprolactone, (Mw ~14,000) (Sigma Aldrich). PVA (Purity 98%) (Merck, Germany). Dicholoromethane (DCM) (Merck, Germany). Sodium hydroxide (NaOH) (Merck, Germany). Potassium bromide (KBr) of FTIR grade (Fischer Scientific UK). Potassium dihydrogen phosphate (KH_2_PO_4_) (Merck, Germany). Hydrochloric acid (HCl) (RDH). Filter paper (Whatman filter paper no. 40) and double distilled water (DDW) was used throughout the studies collected from laboratory.

### 2.2. Preparation of microspheres

MZB loaded floating/hollow microspheres were prepared by oil-in-water (o/w) solvent evaporation technique [25]. Eudragit E 100 and polycaprolactone (PCL) were separately dissolved in dichloromethane (DCM) at 25 °C. They were separately stirred at 300 rpm in separate beakers with a magnetic stirrer on a hot plate. Stirring was continued until a clear solution obtained. Both the solutions were mixed to obtain a homogeneous solution while stirring was continued. MZB was also dissolved in a separate beaker in DCM while stirred at 300 rpm with a magnetic stirrer at 25 °C. When a clear solution obtained of MZB, it was added drop wise in the polymer solution at 300 rpm. Polymer and drug solution stirred to obtain a clear solution. 1% PVA solution was prepared by dissolving PVA at 80 °C on a hot plate at 500 rpm in DDW. Homogeneous solution of drug and polymers was taken in a syringe and added drop wise in 1% PVA solution at 37 °C, while stirring was continued at 800 rpm using magnetic stirrer. Stirring was continued for further 2 h to completely evaporate the DCM. After complete evaporation of DCM, microspheres were filtered on a whatman filter paper and collected. Microspheres that were collected, washed thrice with DDW to remove excess of solvent. Microspheres were dried overnight at room temperature. They were kept in a desiccator after complete drying for further use. Seven formulations were prepared by changing the ratio of polymers. Table 1 indicates the feed composition of synthesized microspheres.

**Table 1.** Feed composition of synthesized Microspheres.

Formulation	Eudragit E100/PCL ratio	Wt. of Eudragit E 100	Wt. of PCL	Drug/Polymer ratio	Concentration of PVA W/V (%)
F1	100/00	1000 mg	–	1:4	1
F2	90/10	900 mg	100 mg	1:4	1
F3	70/30	700 mg	300 mg	1:4	1
F4	50/50	500 mg	500 mg	1:4	1
F5	30/70	300 mg	700 mg	1:4	1
F6	10/90	100 mg	900 mg	1:4	1
F7	00/100	–	1000 mg	1:4	1

### 2.3. Characterization of microspheres

#### 2.3.1. Recovery of microspheres

Recovery or percentage yield of the microspheres is defined as the ratio of weight of microspheres collected or recovered to the total weight of all solid contents taken before the start of the reaction [26]. Collected dried microspheres were weighed to determine the recovery of microspheres:

(1) $$\text{Percentage yield}=\frac{\text{weight of microspheres (mg)}}{\text{weight of all solid species taken at begining (mg)}}\;\times \;100$$

#### 2.3.2. Measurement of microspheres hydration

Microsphere hydration is defined as the ratio between weights of wet microspheres to the weight of dried microspheres. Microspheres recovered weighed immediately at the end of each microencapsulation process and is represented as (M1). When the microspheres are dried to constant weight, they were weighed again and is represented as (M2) [27]. It is represented by the following equation:

(2) $$\%\;\text{Microspheres hydration}=\frac{M1}{M2}\;\times \;100$$

#### 2.3.3. Determination of drug loading

To determine drug loaded in different formulations of microspheres, accurately weighed microspheres were crushed (50 g) and dissolved in specific amount of DCM and diluted it with 0.1 N HCl in a water bath. DCM was removed by agitating the solution at 37 °C. Microspheres were kept for 12 h at 37 °C to dissolve drug completely. Polymers were removed using 0.45 1 m syringe filter. After filtration absorption of MZB clear solution was analyzed at wavelength of 274 nm using UV–vis spectrophotometer (Perkin Elmer) against appropriate blank. Percent drug loading was determined by using following formula [28]:

(3) $$\%\;\text{Drug loading}=\frac{\text{Mass of drug in microspeheres}}{\text{Mass of microspheres}}\;\times \;100$$

For the determination of encapsulation efficiency following formula is used [28]:

(4) $$\%\;\text{Encapsulation efficiency}=\frac{\text{Actual loading of MZB}}{\text{Theoratical loading of MZB}}\;\times \;100$$

### 2.4. Micrometric studies of microspheres

#### 2.4.1. Bulk density

Bulk density can be defined as the total weight of powder divided by the bulk volume. It is not an intrinsic property of powder/granules rather depends on handling of materials. It is measured by pouring the known weight of microspheres in the graduated cylinder and volume of microspheres is determined. It is represented by following formula [29]:

(5) $$\text{Bulk density}=\frac{\text{Sample weight}}{\text{Sample volume}}$$

#### 2.4.2. Tapped density

The packing properties of granules and powder depend on tapped density. Mixing, flow properties of powders/granules and tableting depends on tapped density. To determine the tapped density weighed amount of microspheres were taken in a graduated cylinder and this graduated cylinder is tapped mechanically about 100 times. Volume after 100 tapings was observed. Tapped density can be determined by the following formula [25]:

(6) $$\text{Tapped density}=\frac{\text{weight of microspheres}}{\text{Volume after 100 tappings of microspheres}}$$

#### 2.4.3. Compressibility index

Cohesiveness, size, shape moisture content, surface area and bulk density can be determined using compressibility index or Carr’s index. Actually Carr’s index is used to measure the compressibility of powders. It is calculated by following formula [30]:

(7) $$C_{i}=\frac{\text{Initial volume}-\text{Final volume}}{\text{Initial volume}}\;\times \;100$$

Value of *C*_*i*_ less than 15% represents good flow properties while values of *C*_*i*_ greater than 25% represents poor flow.

#### 2.4.4. Hausner’s ratio

It is named after an engineer Henry H. Hausner. It is another tool to determine the flow ability of granules or microspheres. It is given by following equation:

(8) $${\text{Hausner}}^{\prime }\text{sratio}=\frac{\text{Volume before tapping}}{\text{Volume after tapping}}$$

A value of 1.2 shows free flow and a ratio close to 1 indicate relatively good flow.

#### 2.4.5. Angle of repose

Angle of repose is described as the maximum angle formed between the horizontal plane and surface. It is measured by glass funnel method. Accurately weighed microspheres were passed through a glass funnel on a horizontal plane forming a heap on the surface. The funnel was place in a manner that funnel’s tip was touching the apex/top of heap. It was calculated by following formula [30]:

(9) $$\text{Tan}\theta =\frac{h}{r}$$

where *h* = height of heap; *r* = radius of base cone.

The height of heap was measured by scale and radius was determined by drawing the circles around heap and then measuring the radius.

### 2.5. Equilibrium water uptake study

Blank microspheres prepared by o/w solvent evaporation method were immersed in a buffer solution of pH 1.2 and in a phosphate buffer of pH 7.4 to perform the equilibrium water uptake study. Microspheres were allowed to swell for complete equilibrium study for 24 h at 37 °C. Excess solvents adhered to surface of microspheres was removed using blotting filter paper without exerting any pressure on swollen microspheres. Swelled microspheres were weighed on a single pan balance [31]. Following formula is used for % age equilibrium water uptake:

(10) $$Q\left(\%\right)=\frac{\text{Mass of swollen microspheres}-\text{Mass of dry microspheres}}{\text{Mass of dry microspehers}}\;\times \;100$$

### 2.6. In vitro floating ability studies

MZB loaded floating/hollow microspheres were studied for their floating ability in the simulated gastric medium (0.1 N HCl). Accurately weighed microspheres (50 mg) were taken and spread over the simulated gastric medium present in the type II dissolution test apparatus (Pharmatest). The medium containing microspheres was agitated by paddles at 100 rpm. Temperature was maintained at 37 °C of the medium. They were agitated for 12 h. After 12 h floating and settled at bottom microspheres were taken and dried. After drying both floating and settled microspheres accurately weighed. Percentage of floating microspheres can be calculated by the following formula [32]:

(11) $$\%\;\text{age of floating microspheres}=\frac{\text{weight of floating microsphere after 12 hour}}{\text{Initial weight of floating microspheres}}\;\times \;100$$

### 2.7. Stability study

Accelerated stability studies were performed on the selected formulations as per ICH (The International Conference of Harmonization) guidelines. The optimized formulations was sealed in an aluminum foil and stored at 25 ± 2 °C, 60 ± 5% RH and at 40 ± 2 °C, 75 ± 5% RH for 3 months. Periodically microparticles were removed and evaluated for physicochemical characteristics and *in vitro* drug release [32].

### 2.8. Fourier transform infrared spectroscopy (FT-IR)

Drug-polymer interaction was determined by FT-IR spectroscopy. Potassium bromide (KBr) disc method is used to evaluate drug-polymer interaction. The FT-IR spectra of Eudragit E 100, polycaprolactone, the pure drug (MZB), blank or unloaded microspheres and drug loaded microspheres was taken. Microspheres were crushed to finally grounded state, along with KBr. Hydraulic pressure of 400 KgN^−1^ was applied to prepare disc (2 mg sample in 200 mg KBr).

Scanning range was 4000–400 cm^−1^ and resolution was fixed at 2 cm^−1^ [33].

### 2.9. Scanning electron microscopy (SEM)

SEM was used to study the Morphology and surface characteristics of microspheres. In SEM we studied the surface morphology and topography of the microspheres. SEM analysis of both blank and drug loaded Eudragit microspheres was performed. The samples for the SEM analysis were prepared by sprinkling the small amount of microspheres on one side of the double adhesive stub. The stub was then coated with fine gold dust. The microparticles were then observed with the scanning electron microscope (Leica Electron Optics, Cambridge, USA) at 10 kV, chamber pressure of 0.6 mm Hg and original magnification 500 [32].

### 2.10. In vitro drug release study

The *in vitro* drug release study of MZB loaded floating/hollow microspheres of Eudragit E 100/PCL was performed in dissolution apparatus type II (Pharmatest) according to paddle method described in United States pharmacopoeia.

Accurately weighed microspheres (50 mg) were taken in cellulose dialysis membrane containing sufficient amount of dissolution medium (500 ml) and tied it to paddle. Stirring speed was maintained at 100 rpm while temperature was maintained at 37 °C. 5 ml of release medium was collected with the pippete after fixed intervals of time. An aliquot volume i.e., 5 ml of pre warmed dissolution medium was added to maintain the equal volume. The samples were collected after fixed intervals of time. Drug release was conducted in freshly prepared simulated gastric medium 900 ml and in phosphate buffer of pH 7.4. Samples were tested by measuring the absorption of MZB at 274 nm by using UV–vis spectrophotometer (Perkin Elmer). Drug concentrations were calculated using standard calibration curve [33].

### 2.11. Drug release kinetics

Four kinetic models are more frequently applied to determine the drug release from different controlled release preparations [34]. The *in vitro* drug release data obtained was assessed by the five models to find the best fitting equation.

Zero order release is a system in which drug release is not dependent on concentration of the drug. Equation for zero order release is [35]:

Zero-order kinetics 

(12) $$F_{t}=K_{0}t$$

where *F* indicates the fraction of drug release in time *t* and *K*_0_ is the zero-order release constant.

First-order kinetics [36] 

(13) $$\text{ln}\left(1-F\right)=-K_{1}t$$

where *F* shows the fraction of drug release in time *t* and *K*_1_ is the first-order release constant.

Higuchi model [37] 

(14) $$F=K_{2}t1/2$$

where *F* represents the fraction of drug release in time *t* and *K*_2_ is the Higuchi constant.

Korsmeyer-Peppas model 

(15) $$M_{t}/M_{\infty }=K_{3}t^{n}$$

Here *M*_*t*_ is the amount of drug released in time *t*, *M*_*∝*_ is the amount of drug release at time infinity, *K*_3_ is the kinetic constant and n is the exponent describing the swelling mechanism.

### 2.12. Statistical analysis

Statistical analysis was conducted using one-way ANOVA by SPSS software. Data were displayed as mean  ±  SD and statistical significance was set at *p* <  0.05.

## 3. Results and discussion

### 3.1. Preparation of Eudragit E 100/PCL blend microspheres

In the present study, floating microspheres were prepared loaded with MZB by using oil in water (o/w) solvent evaporation method. This method was selected because drug and both the polymers were soluble in dicholoromethane i.e., oil phase. 1% PVA solution was used as emulsifying agent. Eudragit E 100, PCL and MZB were dissolved in dicholormethane and then introduced in the 1% PVA solution (external phase), stirring speed was kept at 800 rpm for 2 h at 37 °C. Microspheres were recovered as the solvent evaporated. The prepared microspheres were washed to remove excess of solvent three times with water. After washing they were left for drying overnight at room temperature. The formed microspheres were hollow from inside, spherical in shape and white in appearance. Microspheres exhibited good flow properties.

### 3.2. Recovery of microspheres

The recovery of microspheres was increased as the concentration of PCL was increased. Eudragit E 100 is a cationic polymer and was loss in external phase at higher concentration. PCL is a hydrophobic polymer so as the amount of PCL was increased chances of aggregation of microspheres were decreased that resulted in the increase of yield. But when PCL was used alone in the preparation of microspheres, yield was decreased because PCL microspheres were aggregated due to less solvent evaporation and irregular shaped microspheres were formed. Solvent was also not well evaporated which resulted in agglomeration of microspheres. Yield was also affected by stirring speed. During research microspheres were also prepared at 600 rpm and 900 rpm. At low stirring speed obtained microspheres was large in size which formed aggregates and yield was decreased. At higher stirring speed i.e., 900 rpm, small and irregular shaped microspheres were prepared which also formed aggregates that decreased the yield [38]. Table 2 indicates the % recovery of microspheres.

**Table 2.** Recovery % of microspheres.

Formulation	Mean input of all solid contents (mg)	Mean output (mg)	% Recovery of microspheres
F1	1250	806	64.48
F2	1250	863	69.04
F3	1250	882	70.56
F4	1250	901	72.08
F5	1250	912	72.96
F6	1250	887	68.08
F7	1250	773	61.84

Note: Data indicates the mean ± standard deviations are representative of at least three different experiments.

### 3.3. Degree of hydration of microspheres

Degree of hydration depends on the hydrophilicity of the polymers. A polymer which is hydrophilic in nature swells more in water and hydrophobic polymer swells the least. PCL is a hydrophobic polymer so by increasing the concentration of PCL the degree of hydration was decreased because very small amount of water was retained in the microspheres. Eudragit E 100 is a polymer that is soluble at pH less than 5 and swells in water. Degree of hydration increased when the concentration of Eudragit E 100 was raised. Drug release pattern was also affected by the degree of hydration. Drug release will be hindered considerably if the aqueous medium does not penetrate in to the polymeric matrix [39]. Table 3 shows the % microspheres hydration.

**Table 3.** %Microspheres Hydration.

Formulation	Mean weight of wet microspheres (mg)	Mean weight of dry microspheres (mg)	Microsphere hydration (%)
F1	1381	806	171.33
F2	1408	863	163.15
F3	1367	882	154.98
F4	1316	901	146.05
F5	1317	912	144.40
F6	1224	887	137.99
F7	1022	773	132.21

Note: Data indicates the mean ± standard deviations are representative of at least three different experiments.

### 3.4. Rheological parameters of microspheres

Rheological studies included bulk density, tapped density, compressibility index or Carr’s index (*C*_*i*_), Hausner’s ratio (*H*_*r*_) and angle of repose. All the seven formulations were studied for all the properties. Table 4 indicates the values of *C*_*i*_ of all seven formulations. *C*_*i*_ values lies between 11 and 18 which showed an excellent flow of microspheres. *H*_*r*_ values of all seven formulations are below 1.25 indicating good flow properties. Values of angle of repose of all formulations are below 30° also indicating free flow properties of microspheres. Similar findings are reported by. [39] Table 4 indicates the rheological parameters of synthesized microspheres.

**Table 4.** Rheological studies of microspheres.

Formulation	Bulk density	Tapped density	Compressibility index	Hausner’s ratio	Angle of repose
F1	0.17	0.20	13.63	1.15	12.04
F2	0.16	0.19	16	1.15	11.58
F3	0.23	0.29	13.33	1.15	12.46
F4	0.21	0.26	17.54	1.21	11.23
F5	0.24	0.27	11.76	1.13	15.6
F6	0.27	0.31	12.5	1.14	11.91
F7	0.20	0.25	17.94	1.21	12.33

Note: Data indicates the mean ± standard deviations are representative of at least three different experiments.

### 3.5. Drug loading and encapsulation efficiency of microspheres

Effect of varying polymer ratio was investigated on the drug loading and encapsulation efficiency of the microspheres. The % drug loading and encapsulation efficiency is reported in Tables 5 and 6 respectively. It was observed that drug loading was increased as the amount of Eudragit E 100 increased. The reason was formation of hollow microspheres having maximum amount of pores that help in more loading of drug. Encapsulation efficiency found high when the ratio of Eudragit E 100 and PCL was at 50:50 and went on decreasing by changing either of the concentration of Eudragit E 100 and PCL. When concentration of PCL was increased more than 50% drug loading and encapsulation efficiency started decreasing. The reason was hydrophobic nature of the PCL that formed thick wall microspheres having less pores in the microspheres.

**Table 5.** Percent drug loading in microspheres.

Formulation	Mass of microspheres (mg)	Mass of drug in microspheres (mg)	% Drug loading
F1	50	4.121	8.242 ± 0.40
F2	50	4.499	8.998 ± 0.34
F3	50	4.837	9.674 ± 0.22
F4	50	5.015	10.03 ± 0.05
F5	50	3.968	7.936 ± 0.46
F6	50	3.889	7.778 ± 0.39
F7	50	3.720	7.44 ± 0.27

**Table 6.** Encapsulation efficiency of microspheres.

Formulation	Theoretical loading (mg)	Actual loading (mg)	Encapsulation efficiency (%)
F1	250	65.93	26.37 ± 1.35
F2	250	76.48	30.59 ± 0.98
F3	250	84.64	33.85 ± 1.28
F4	250	87.79	35.11 ± 1.51
F5	250	73.32	29.32 ± 0.99
F6	250	67.97	27.18 ± 1.39
F7	250	57.66	23.06 ± 1.77

### 3.6. In vitro evaluation of floating ability of microspheres

Buoyancy or floating ability of hollow microspheres was checked in simulated gastric fluid i.e., pH 1.2. It was observed that after 12 h maximum floating ability was observed in F7 because PCL is a hydrophobic polymer and does not dissolved in gastric fluid. As the concentration of Eudragit E 100 was increased floating ability of microspheres decrease. Eudragit E100 microspheres were soluble in the simulated gastric fluid because of nature of polymer. All the seven formulations have floating ability between 60 and 85%. Table 7 indicates the buoyancy or floating ability of hollow microspheres in gastric medium.

**Table 7.** % Floating ability of microspheres at pH 1.2.

Formulation	Weight of microspheres taken initially(mg)	Weight of floating microspheres (mg)	% Floating ability
F1	50	30.6	61.2
F2	50	33.7	67.4
F3	50	35.3	70.6
F4	50	37.9	75.8
F5	50	39.8	79.6
F6	50	41.2	82.4
F7	50	42.5	85

Note: Data indicates the mean ± standard deviations are representative of at least three different experiments.

### 3.7. Equilibrium water uptake studies

Eudragit E 100 is a polymer that is soluble at pH below 5. This polymer has higher water uptake ability at pH 1.2. While the PCL is a strongly hydrophobic polymer it does not absorb large amount of water. When all seven formulations analyzed it was revealed that by increasing amount of PCL, water uptake of microspheres decreased in lower pH solution (1.2). However an increase in water uptake was observed for microspheres in solution of high pH (7.4) with increasing PCL ratio. While when concentration of Eudragit E 100 was increased water uptake by microspheres also increased.

### 3.8. In vitro drug release studies

**Figure 2.**
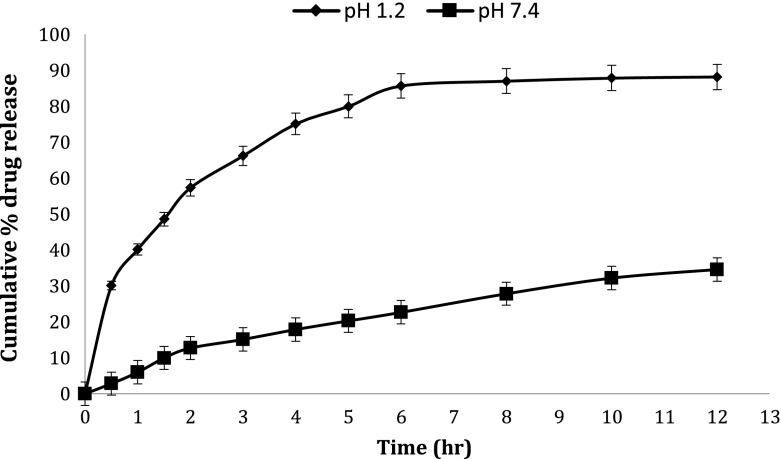
% cumulative drug release from Eudragit E 100 microspheres (100/00) using PVA as an emulsifier (1%) and 0.250 g of MZB at pH 1.2 (♦) and pH 7.4(■).

**Figure 3.**
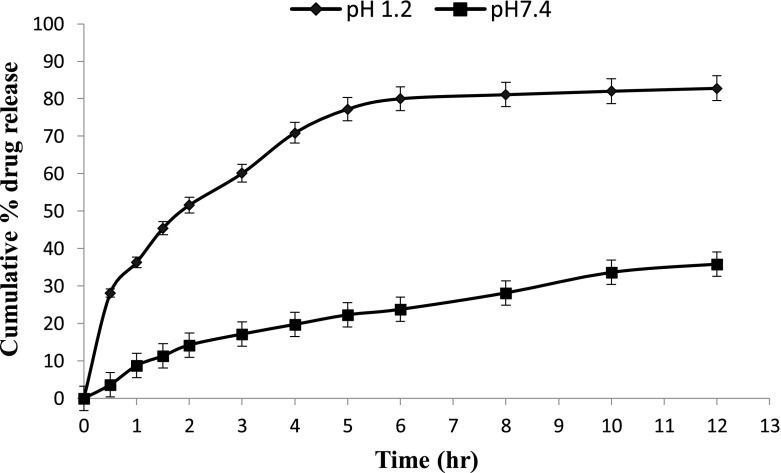
% cumulative drug release from Eudragit E 100/PCL microspheres (90/10) using PVA as an emulsifier (1%) and 0.250 g of MZB at pH 1.2 (♦) and pH 7.4(■).

**Figure 4.**
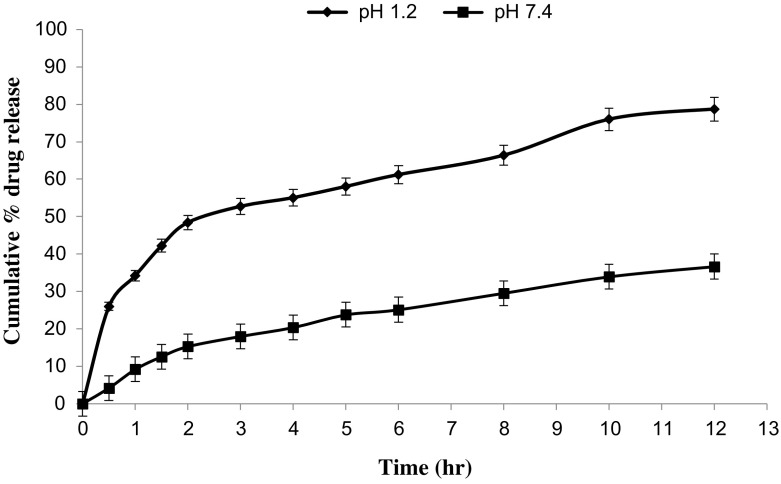
% cumulative drug release from Eudragit E 100/PCL microspheres (70/30) using PVA as an emulsifier (1%) and 0.250 g of MZB at pH 1.2 (♦) and pH 7.4(■).

**Figure 5.**
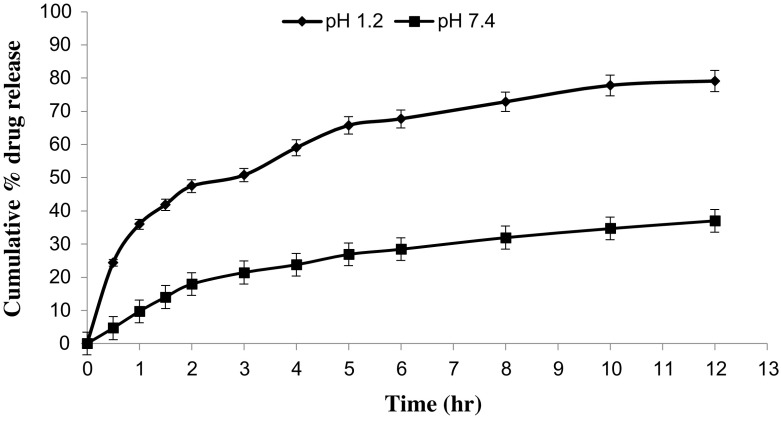
% cumulative drug release from Eudragit E 100/PCL microspheres (50/50) using PVA as an emulsifier (1%) and 0.250 g of MZB at pH 1.2 (♦) and pH 7.4(■).

**Figure 6.**
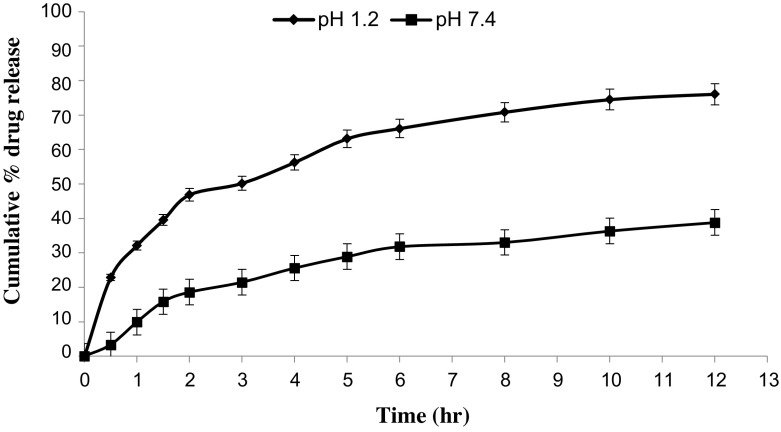
% cumulative drug release from Eudragit E 100/PCL microspheres (30/70) using PVA as an emulsifier (1%) and 0.250 g of MZB at pH 1.2 (♦) and pH 7.4(■).

**Figure 7.**
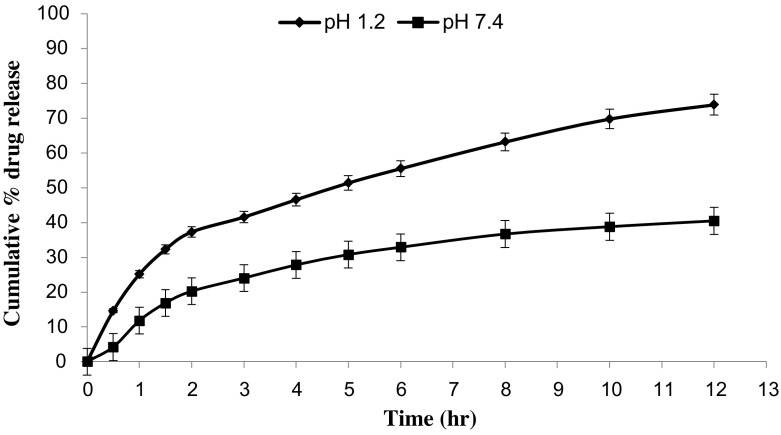
% cumulative drug release from Eudragit E 100/PCL microspheres (10/90) using PVA as an emulsifier (1%) and 0.250 g of MZB at pH 1.2 (♦) and pH 7.4(■).

**Figure 8.**
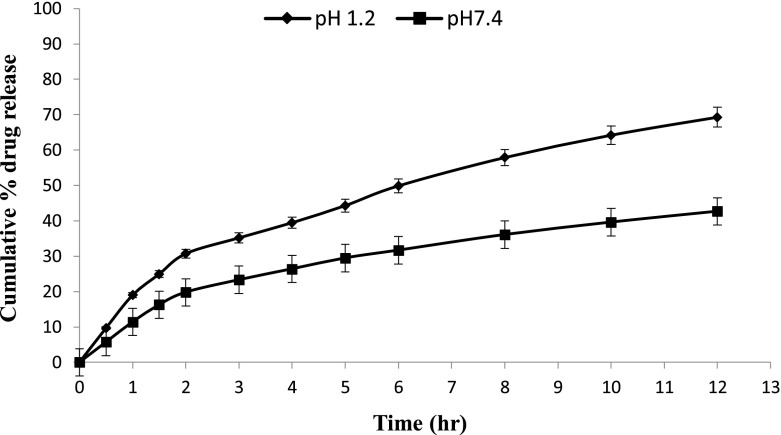
% cumulative drug release from PCL microspheres (00/100) using PVA as an emulsifier (1%) and 0.250 g of MZB at pH 1.2 (♦) and pH 7.4(■).

**Figure 9.**
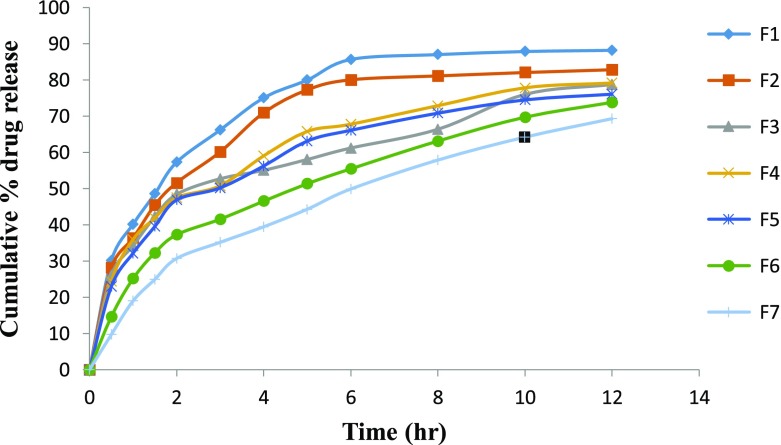
% cumulative drug release from all 7 formulations of Eudragit E 100/PCL microspheres using PVA as an emulsifier (1%) and 0.250 g of MZB at pH 1.2.

**Figure 10.**
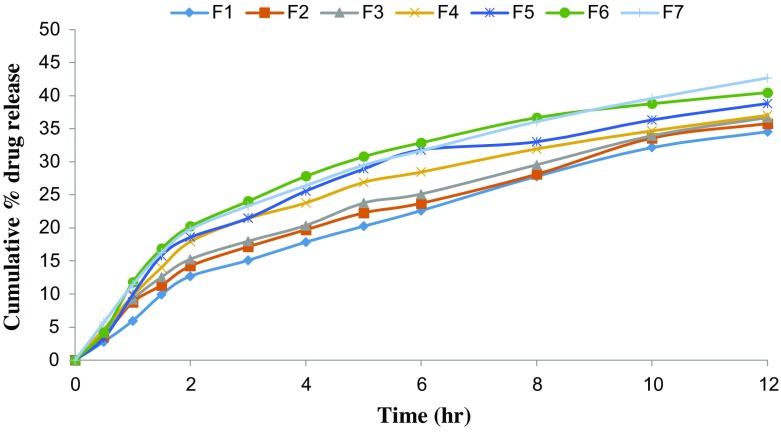
% cumulative drug release from all 7 formulations of Eudragit E 100/PCL microspheres using PVA as an emulsifier (1%) and 0.250 g of MZB at pH 7.4.

**Figure 11.**
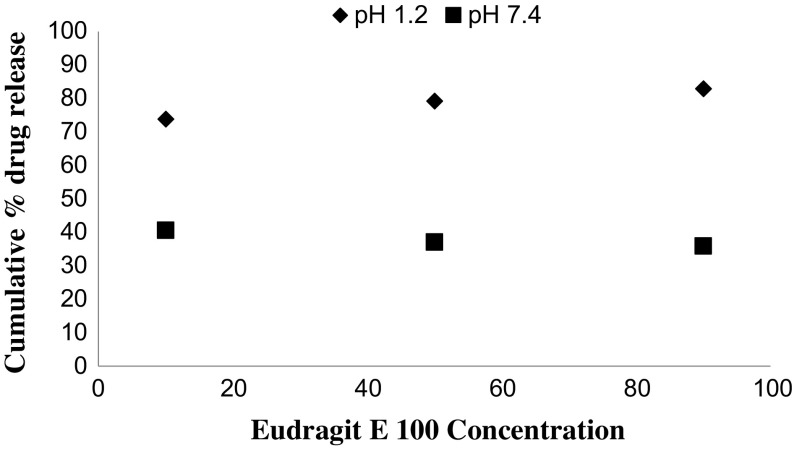
% release of MZB from Eudragit E 100/ PCL microspheres with concentration of Eudragit E 100 (10, 50 and 90%) at pH 1.2 and 7.4.

**Figure 12.**
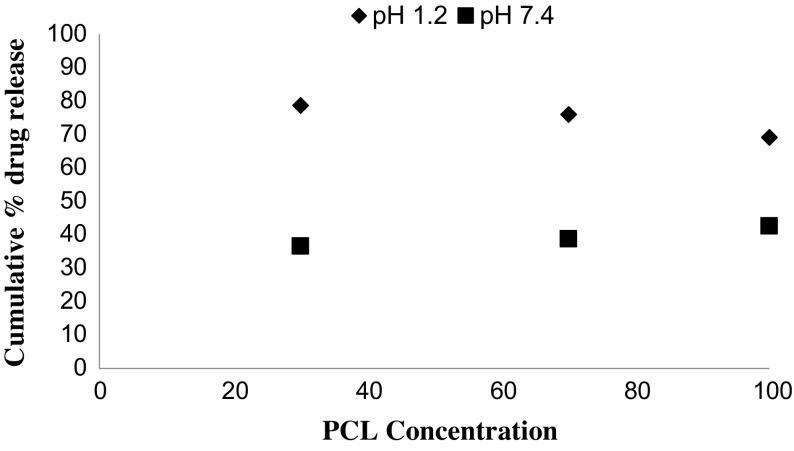
% release of MZB from Eudragit E 100/ PCL microspheres with concentration of PCL (30, 70 and 100%) at pH 1.2 and 7.4.

*In vitro* drug release studies were performed in the simulated gastric fluid i.e., pH 1.2 and in simulated intestinal fluid i.e., phosphate buffer of pH 7.4. In simulated gastric fluid, it was observed that drug released in a sustained manner. From formulation containing higher concentrations of Eudragit E 100, 50% or more drug released within the first two hours i.e., in burst release manner. As the concentration of PCL increased in the microspheres, drug was released in a continuous fashion. Same results were reported by Jeong et al. in their work [40]. It was because Eudragit E 100 microspheres were more porous and drug rapidly released from them. From F1 to F2 containing higher amounts of Eudragit E 100, 80% of drug released during first six hours. Another reason for abrupt release of drug from formulations containing higher amount of Eudragit E 100 was its solubility under pH 5. Figures 2–8 indicates the *invitro* drug release at variable polymeric concentrations in different medias. The other five formulations released the drug in a sustained release pattern up to 12 h. When the concentration of Eudragit E 100 was decreased or absent in a formulation, drug release also retarded. Figures 9 and 10 refers to *in vitro* drug release of all formulations in buffer solutions of different pH values. *Invitro* drug release at pH 7.4 was very low because of nature of the polymers and microspheres. Eudragit E 100 have less swelling ability above pH 5 that’s why could not release the drug in a sustained manner. Due to less floating ability of microspheres at pH 7.4, hence they could not release the drug at desired release rate. However drug release at pH 7.4 was observed due to the presence of PCL contents in the feed composition owing to their highest water uptake. Formulation F4 (50:50) showed good and sustained release pattern as compared to other ratios in both mediums, because in solution of lower pH (1.2), Eudragit E100 has good solubility, which leads to the release of encapsulated drug, while in solution of higher pH (7.4), PCL has highest swelling due to increased water uptake. This in turn leads to increased drug release at higher pH (7.4). Figures 11 and 12 indicates the effect of polymeric concentration on cumulative % drug release.

### 3.9. Drug-release-kinetics

*In vitro* drug release data was analyzed by applying different release kinetic models i.e., zero order, first order, Higuchi and Korsemeyer Peppas model. The drug release constants (*k*, *r*, *n*) were also calculated. The high value of (*r*) was used for all seven formulations. Drug release pattern was swelling of the matrix of polymers. The buffer entered into blend microspheres, dissolved the drug and come outside the microspheres. By applying Higuchi model the value of *r* was calculated which showed diffusion controlled drug release pattern. It was then plotted between square root of time and drug release in a graph. By applying Korsemeyer Peppas model the value of n for release of MZB was calculated by intercept and slope of graph. In all trials, drug release mechanism was studied by applying Korsmeyer Peppas model, n values in first five formulations are less than 0.45 indicating fickian release. The value of n of all seven formulation at pH 7.4 was between 0.45 and 1 which indicated that diffusion mechanism was non fickian with the swelling of polymer. It was found that *in vitro* drug release of all formulation at pH 1.2 and pH 7.4 was best explained by Higuchi release equation. It showed highest linearity as shown in Tables 8 and 9 suggesting release of drug from insoluble matrix by time dependent diffusion process based on Fick’s law.

**Table 8.** Release kinetics of drug at different pH from microspheres.

Formulation	pH	Zero order release kinetics		First order release kinetics		Higuchi model	
		*K*_0_ (h^−1^)	*r*	*K*_1_ (h^−1^)	*r*	*K*_2_ (h^−1^)	*r*
F1	1.2	4.749	0.752	0.165	0.873	0.219	0.890
	7.4	2.565	0.959	0.045	0.972	0.133	0.994
F2	1.2	4.537	0.749	0.131	0.841	0.209	0.886
	7.4	2.590	0.952	0.038	0.951	0.127	0.988
F3	1.2	4.057	0.903	0.100	0.971	0.178	0.970
	7.4	2.601	0.944	0.038	0.954	0.128	0.990
F4	1.2	4.375	0.865	0.110	0.956	0.195	0.961
	7.4	2.551	0.882	0.037	0.908	0.055	0.964
F5	1.2	4.275	0.851	0.100	0.941	0.192	0.954
	7.4	2.726	0.853	0.039	0.866	0.131	0.937
F6	1.2	4.671	0.923	0.097	0.986	0.204	0.939
	7.4	2.807	0.848	0.042	0.874	0.137	0.986
F7	1.2	4.801	0.942	0.089	0.990	0.209	0.993
	7.4	2.921	0.910	0.045	0.927	0.142	0.973

**Table 9.** Drug release mechanism from all formulations at different pH values.

Formulation	pH	Korsmeyer-peppas model		Order of release
		Release exponent (*n*)	*R*	
F1	1.2	0.354	0.952	fickian
	7.4	0.803	0.972	Non-fickian
F2	1.2	0.362	0.952	fickian
	7.4	0.699	0.967	Non-fickian
F3	1.2	0.329	0.978	fickian
	7.4	0.670	0.967	Non-fickian
F4	1.2	0.361	0.979	fickian
	7.4	0.756	0.915	Non-fickian
F5	1.2	0.375	0.976	fickian
	7.4	0.709	0.890	Non-fickian
F6	1.2	0.473	0.975	Non-fickian
	7.4	0.661	0.902	Non-fickian
F7	1.2	0.575	0.973	Non-fickian
	7.4	0.620	0.958	Non-fickian

### 3.10. Accelerated stability studies

The prepared optimized formulations were selected to perform the accelerate stability studies at designated conditions (25 ± 2 °C, 60 ± 5% RH and at 40 ± 2 °C, 75 ± 5% RH). The formulations were stored for 3 months. After 3 months storage, formulations were evaluated to check the values of all parameters like % drug content, % encapsulation efficiency and found to be almost similar to the initial values. The drug release profile was also found similar to the initial profile. It was observed that there is no significant change in the physical and chemical properties after the accelerated stability studies.

### 3.11. FTIR analysis

The pure drug and optimized formulations were subjected for FTIR analysis. The samples were scanned over a range of 4000–400 cm^−1^.

**Figure 13.**
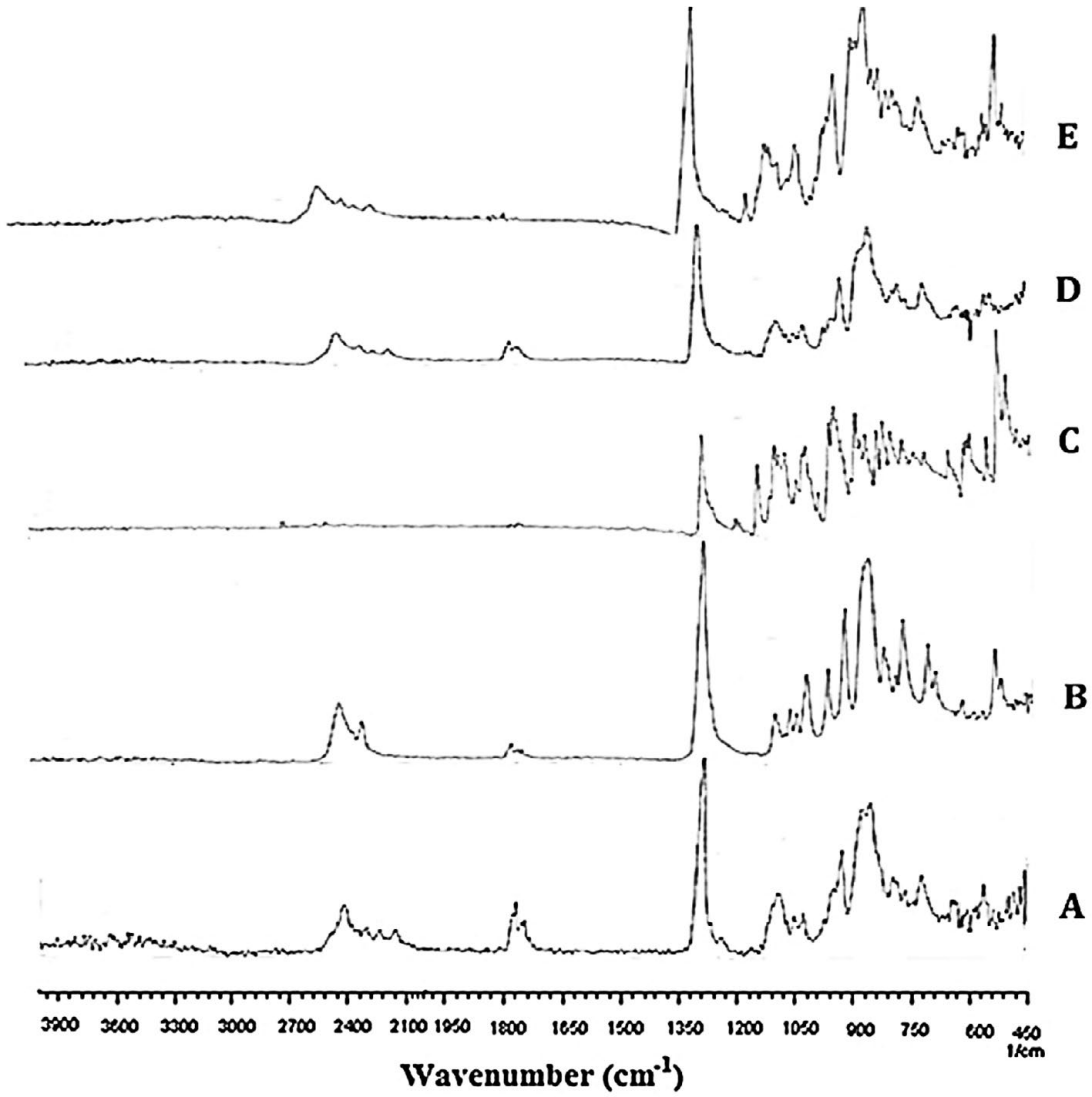
FTIR spectra of pure Eudragit E 100 (a), PCL (b) MBZ (c) blank microspheres (d) and drug loaded microspheres (e).

Figure 13 shows the FTIR spectra of pure Eudragit E 100 (a), PCL (b) MBZ (c) blank microspheres (d) and drug loaded microspheres (e). The FTIR spectra of pure Eudragit E 100 i.e., Figure 13(a) showed an ester C = O stretching peak around 1799.48 cm^−1^. In the FTIR spectra of pure PCL shown in Figure 13(b), the peak at 1775 cm^−1^is attributed to the carbonyl group stretching absorption. While the prominent characteristic peaks at 2589 cm^−1^ show the stretching of OH group of carboxylic acid. Two peaks in the range of 1280–1378 cm^−1^ are related to carboxylate C = O stretching. The peaks in the range of 750–1075 cm^−1^ are attributed to stretching of CH_2_ and OH group, respectively. The FTIR spectra of pure drug i.e., Figure 13(c) show characteristic peaks at (2719 cm^−1^) OH stretch, (1495 cm^−1^) N–O stretch, (974–1045 cm^−1^) C–OH, C–O stretch, C–NO, and (875 cm^−1^) C–N stretch. FTIR analysis of the prepared blank microspheres was performed and results obtained are presented in Figure 13(d). FTIR spectra of native blank microspheres show a new absorption band in the range of 1650–1800 cm^−1^. This peak is attributed to -NH bond stretching vibrations. FTIR spectra of drug loaded microspheres shown in Figure 13(e) reveal no significant changes as compared to blank microspheres. The characteristics of the peak of drug (metronidazole) were not altered after encapsulation, which shows that there is no chemical interaction between drug and polymer.

### 3.12. SEM analysis

**Figure 14.**
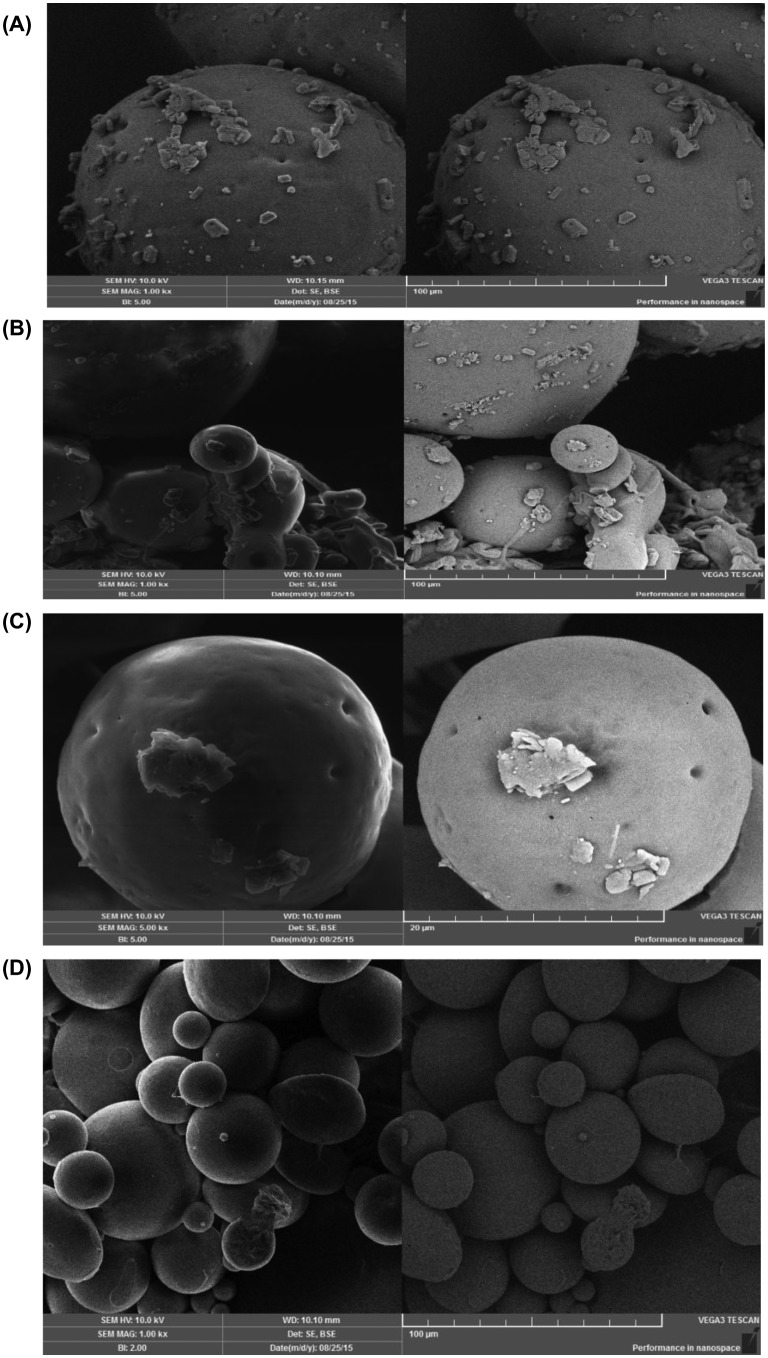
Scanning electron micrographs of synthesized microspheres at various resolutions.

SEM study reveal that prepared microspheres are spherical in shape as shown in Figure 14. The microspheres seems to be more spherical in shape with smooth surfaces i.e., Figure 14(c) while some smaller particles showed wrinkled morphology i.e., Figure 14(a). The wrinkled morphology of the particles suggest that the shell formed during the early stages of the droplet formation is not so rigid.

The surface of the drug-loaded Eudragit E 100/PCL (70/30) microspheres show the presence of drug particles on their surface as shown in Figure 14(a), (b) and (d). All the microspheres also showed small pores on their surfaces that as shown in Figure 14(c). These pores believed to facilitate the diffusion of solvent into the shell of microparticles as well as release of the drug out of the particle matrix.

## 4. Conclusion

PCL/Eudragit E 100 blend microspheres loaded with MZB were formulated successfully by using o/w solvent evaporation method. Recovery of microspheres, degree of hydration, % drug loading, % encapsulation efficiency, *in vitro* floating ability were affected by changing the ratio of polymers. Rheological properties indicated the prepared microspheres were free flowing. *In vitro* floating ability test showed that microspheres were hollow and floating above the surface of simulated gastric fluid. *In vitro* drug release studies revealed that microspheres were able to release the drug up to 12 h in the simulated gastric fluid. It was observed that almost 70% or above drug released from all formulations. Formulation F4 released the drug in a continuous sustained manner. Formulations containing high amount of Eudragit E 100 released the drug in a rather abrupt release manner in the beginning. Formulation F4 which had the PCL/Eudragit E 100 ratio (50:50) was found to be best releasing in both mediums. Drug release kinetics followed Higuchi model at pH 1.2 and 7.4. Drug release pattern was non-fickian for all formulations at pH 7.4 while F6 and F7 at pH 1.2 also followed non fickian mechanism. Formulations (F1 to F5) at pH 1.2 followed fickian release mechanism. FTIR spectroscopy confirmed the presence of respective functional groups in pure polymers and synthesized microspheres. SEM study showed the porous nature of the synthesized microspheres that facilitate the diffusion of solvent and solute in and out of the microspheres.

## Disclosure statement

No potential conflict of interest was reported by the authors.
